# Maternal inheritance and mitochondrial DNA variants in familial Parkinson's disease

**DOI:** 10.1186/1471-2350-11-53

**Published:** 2010-04-01

**Authors:** David K Simon, Nathan Pankratz, Diane K Kissell, Michael W Pauciulo, Cheryl A Halter, Alice Rudolph, Ronald F Pfeiffer, William C Nichols, Tatiana Foroud, the Parkinson Study Group - PROGENI Investigators

**Affiliations:** 1 Department of Neurology; Beth Israel Deaconess Medical Center and Harvard Medical School, Boston, MA, USA; 2 Department of Medical and Molecular Genetics, Indiana University Medical Center, Indianapolis, Indiana, USA; 3 Division of Human Genetics, Cincinnati Children's Hospital Research Center, Cincinnati, OH, USA; 4 Department of Neurology, University of Rochester School of Medicine, Rochester, NY, USA; 5 Department of Neurology, University of Tennessee School of Medicine, Memphis, TN, USA; 6 Department of Pediatrics, University of Cincinnati School of Medicine, Cincinnati, OH, USA

## Abstract

**Background:**

Mitochondrial function is impaired in Parkinson's disease (PD) and may contribute to the pathogenesis of PD, but the causes of mitochondrial impairment in PD are unknown. Mitochondrial dysfunction is recapitulated in cell lines expressing mitochondrial DNA (mtDNA) from PD patients, implicating mtDNA variants or mutations, though the role of mtDNA variants or mutations in PD risk remains unclear. We investigated the potential contribution of mtDNA variants or mutations to the risk of PD.

**Methods:**

We examined the possibility of a maternal inheritance bias as well as the association between mitochondrial haplogroups and maternal inheritance and disease risk in a case-control study of 168 multiplex PD families in which the proband and one parent were diagnosed with PD. 2-tailed Fisher Exact Tests and McNemar's tests were used to compare allele frequencies, and a t-test to compare ages of onset.

**Results:**

The frequency of affected mothers of the proband with PD (83/167, 49.4%) was not significantly different from the frequency of affected females of the proband generation (115/259, 44.4%) (Odds Ratio 1.22; 95%CI 0.83 - 1.81). After correcting for multiple tests, there were no significant differences in the frequencies of mitochondrial haplogroups or of the 10398G complex I gene polymorphism in PD patients compared to controls, and no significant associations with age of onset of PD. Mitochondrial haplogroup and 10398G polymorphism frequencies were similar in probands having an affected father as compared to probands having an affected mother.

**Conclusions:**

These data fail to demonstrate a bias towards maternal inheritance in familial PD. Consistent with this, we find no association of common haplogroup-defining mtDNA variants or for the 10398G variant with the risk of PD. However, these data do not exclude a role for mtDNA variants in other populations, and it remains possible that other inherited mitochondrial DNA variants, or somatic mDNA mutations, contribute to the risk of familial PD.

## Background

Mitochondrial complex I is impaired in platelets [1] and in the substantia nigra [2] in PD. Inhibitors of complex I induce parkinsonism in a variety of animal models [3,4], suggesting that complex I deficiency may contribute to the pathogenesis of PD. Evidence in support of the possibility that mtDNA variants may play a role in PD is the reported bias towards maternal inheritance of PD [5,6], though a maternal bias has not been detected in all studies [7,8]. Additional evidence comes from studies of cell lines expressing mtDNA from PD patients, which recapitulate the complex I defect found in PD, suggesting that mtDNA mutations account for the complex I dysfunction [9-11]. A multigenerational family has been reported with maternally inherited PD associated with a mitochondrial complex I defect in cybrids created from affected family members, although specific mtDNA mutations were not reported[11]. Other rare families with parkinsonism associated with mtDNA point mutations or multiple mtDNA deletions have been reported [12-18]; however, extensive searches for mtDNA mutations in PD patients have failed to reveal clearly pathogenic mutations in the vast majority of patients [19-21]. Heteroplasmic mitochondrial *ND5 *gene mutations have been associated with PD [22,23], but these mutations were present at very low levels and so are unlikely to be of functional significance. Certain mitochondrial haplogroups have been reported to be associated with the risk of PD, but these studies have yielded variable results [24-31]. A common variant in a mitochondrial complex I gene, 10398G, has been reported to be less frequent in PD patients, suggesting a protective effect [25,32]; however, this has not been consistently confirmed across studies [19,24]. Thus, the contribution of mtDNA variants to PD risk remains unclear. Furthermore, prior studies have not addressed the possibility of an association of mtDNA variants with other clinical features, such as age of onset of PD.

We sought to investigate the association of mtDNA variants with the risk of PD and with the age of onset of PD by taking advantage of a unique cohort of familial PD subjects. We focused on the subset of families in which the proband and either the mother or the father were diagnosed with PD. This allowed us to test for a maternal bias in the inheritance of PD and also to test whether any of the common mtDNA haplotypes are risk factors for disease.

## Methods

### Subjects

As part of an ongoing genetic study recruiting siblings diagnosed with PD, 654 multiplex PD families were ascertained. All available affected individuals were seen by a movement disorder specialist at one of 59 Parkinson Study Group sites. A standardized clinical assessment was completed including the validated Unified Parkinson's Disease Rating Scale (UPDRS) Parts II & III [33]. A diagnostic checklist with inclusion and exclusion criteria based on previously described clinicopathological correlates [34] was used as previously described [35]. Peripheral blood for DNA extraction was obtained from all individuals after obtaining written informed consent approved by each institution's review board.

To explore the role of inherited mitochondrial factors, a subset of families was analyzed which included a proband with PD that was verified by direct clinical examination and with either a mother or a father (but not both) diagnosed with PD (n = 168). These 168 families excluded families with either two *parkin *mutations (homozygous or compound heterozygous) or a *LRRK2 *mutation. Families in which both the mother and the father were diagnosed with PD were also excluded from analyses (n = 11).

**Figure 1:**
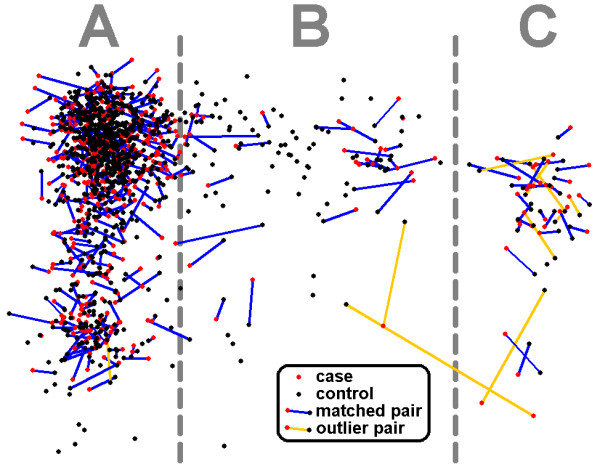
**Case-control pairings**. All 488 PROGENI cases that were recently included in a genomewide association study [37] were matched to a control by minimizing the distance between each case-control pair in four dimensional space (age, gender, and two ethnicity components). Figure 1 shows the pairs in two dimensional space (just the ethnicity components). Those with pairings with 4-dimensional distances greater than three standard deviations from the mean were not included in the analyses (color coded in orange). Based on those samples where religion and country of origin was known to be homogeneous for all four grandparents (~15% of samples), we have inferred that those samples in region A represent Northwestern European ancestry, region B represents Southern European and the Middle Eastern ancestry, and region C represents Ashkenazi Jewish ancestry. The significance of the ethnicity component plotted on the Y axis did not follow a clear recognizable pattern.

Control samples (n = 895) were obtained from the NINDS Human Genetics Resource Center at the Coriell Institute Coriell Cell Repositories (Camden, NJ). These control samples as well as a single individual from each of 488 of the Parkinson's Research: The Organized Genetics Initiative (PROGENI) families were genotyped at the Center for Inherited Disease Research (CIDR) using the Illumina HumanCNV370 version1_C BeadChips (Illumina, San Diego, CA, USA) and the Illumina Infinium II assay protocol[36] as part of a study to identify genetic risk factors contributing to PD susceptibility [37]. This genome-wide data was then used to match cases with controls based on age, gender, and ethnicity as determined by the multidimensional scaling (MDS) algorithm implemented in PLINK [38]. As described previously [37], a MDS analysis identified individuals with African, Asian or Hispanic decent. These individuals were removed to avoid confounding issues of population stratification, and the MDS analyses were repeated using only those individuals who were both self-declared to be non-Hispanic Caucasian and who clustered as non-Hispanic Caucasians. The first two components from this second MDS analysis yielded one large cluster with Northwestern European ancestry, a smaller cluster with Ashkenazi Jewish ancestry, and a continuum of individuals in between with an origin from Southern Europe and the Middle East (see Figure 1). It is these first two components that were used to match cases with controls.

Cases and controls were matched in four dimensional space (gender, age, and two MDS components) using the following algorithm. Gender was coded as 0 and 1, the age distribution was normalized and multiplied by 4, and each of the first two MDS components were normalized and multiplied by 8. The Euclidean distance between all possible cases and all possible controls was then computed, and the minimum distance to a control was determined for each case. The case-control pair with the minimum distance was flagged and declared a final match. The selection process was then repeated iteratively with the remaining individuals, flagging the pair with the minimum distance, until all cases were matched to a control. The distribution of distances between cases and their final matched control sample was reviewed and those pairs that were beyond 3 standard deviations from the mean were not included in the paired analyses (n = 10). The weights used (1, 4 and 8) were selected to balance the contribution of each factor and minimize the number of outliers; without these weights, gender dominated the matching process at the expense of the ethnicity components. Demographics of cases and controls are indicated in Table 1.

**Table 1:** Demographics

	Cases	Controls
N	468	468

Male	60%	55%

Age at onset (cases) or exam (controls)	61.8 ± 10.2 (range: 21-84)	60.7 ± 11.0 (range: 25-86)

Non-Hispanic Caucasian	100%	100%

### Genotyping

To allow haplogroup identification, 12 SNPs were genotyped. These included 10 SNPs genotyped in previous reports that are associated with particular haplogroups [32]: 1719A (Haplogroups I and X), 4580A (Haplogroup V), 7028C (Haplogroup H), 8251A (Haplogroups I and W), 9055A (Haplogroup K), 10398G (Haplogroups I, J, and K), 12308G (Haplogroups K and U), 13368A (Haplogroup T), 13708A (Haplogroup J), and 16391A (Haplogroup I), as well as two additional SNPs (10400T and 16390A) that were included to reduce the number of ambiguous haplogroups. Genotyping was performed by use of custom TaqMan SNP Genotyping assays (Applied Biosystems, Foster City, CA) and restriction digests. Taqman assays were designed using ABI's Custom Taqman Assay Design Tool. SNPs for which Taqman assays were used (1719A, 4580A, 7028C, 9055A, 12308G, 13368A, 13708A) were assayed using 30 ng of genomic DNA and ABI's Taqman Genotyping Master Mix. The assays were run on thermocyclers according to manufacturer instructions and post-read on a 7300 Real Time PCR System using SDS software version 1.3.1 (Applied Biosystems, Foster City, CA). SNPs for which restriction digests were used (8251A, 10398G, 16391A) were assayed by using 40 ng of genomic DNA for PCR amplification followed by restriction digest with enzymes HaeIII (8251A), DdeI/AluI (10398G), or BamHI/HinfI (16391A) according to manufacturer instructions (New England Biolabs, Ipswich, MA). Restriction digest products were electrophoresed through a 4% Genepure Sieve GQA agarose gel (ISC BioExpress, Kaysville, UT) and visualized by ethidium bromide staining. The two additional SNPs assayed (10400T and 16390A) were assayed by Taqman as described above. Because some SNPs do not tag their haplotypes exclusively, alleles that were seen in less than 95% of a reference panel of 560 individuals [39] were not required for their respective haplotypes. These include 8251A for haplogroup I, 10398G for haplogroups I/J/K, and the major allele 13708G for haplogroup X (Table 2). All haplogroups could still be unambiguously defined when there was no missing data. Individuals that had one or more missing genotype and their matching case/control were not included in analysis (n = 10 pairs).

**Table 2:** Classification of Haplogroups

Haplo-group	1719	4580	7028	8251	9055	10398	10400	12308	13368	13708	16391
H	99% G	G	95% C	G	99.6% A	A	C	A	G	98% A	G

I	A	G	T	**93% A**	G	**93% G**	C	A	G	G	A

J	97% G	G	T	G	G	**88% G**	C	A	G	A	G

K	G	G	T	G	A	**70% G**	C	G	G	G	G

T	98% G	G	T	G	G	A	C	A	A	G	G

U	G	G	T	G	G	A	C	G	G	98% A	G

V	G	A	T	G	G	A	C	A	G	G	G

W	G	G	T	A	G	A	C	A	G	G	G

X	A	G	T	G	G	A	C	A	G	**45% A**	G

M	G	G	T	G	G	G	T	A	G	G	G

Those tag SNPs present on less than 95% of reference haplotypes [39] are listed in **bold **and were not required to define the corresponding haplogroup. Given no missing data at the other loci, haplogroups could still be unambiguously defined in all instances.

### Statistical methods

Proportions of male versus female PD subjects in the parent versus offspring generations were compared using a 2-tailed Fisher's exact test. A 2-tailed Fisher's exact test was also used for comparison of the frequencies of mitochondrial SNP and haplogroups between PD cases and controls, and also for comparison of the mitochondrial SNP and haplogroup frequencies between PD cases with an affected mother and those with an affected father. In order to take advantage of the paired study design, the data also were analyzed using the more powerful McNemar's test. In this test the number of discordant matched pairs are determined, such that b = the number of pairs where the case is a carrier of the variant and the matched control is not and c = the number of pairs where the case is not a carrier and the control is. If b is significantly greater than c, then the null hypothesis is rejected, suggesting that the allele increases risk for disease. When b+c < 25, then significance (the p-value) is determined by the sign test (binomial distribution) instead of McNemar's test.

Age at onset was compared between carriers and non carriers of each variant using an independent t-test. Since all siblings share the same mitochondrial SNP alleles, and therefore haplogroups, we repeated these analyses substituting the mean age at onset of all affected siblings in a nuclear family for the age at onset of the individual genotyped. The number of affected siblings in a family ranged from 1 to 5.

## Results

A total of 168 families were identified in which one parent and one or more offspring were affected with PD, with at least one offspring having had their PD diagnosis verified by direct clinical examination. There were 259 siblings among the offspring generation of these families, of which 115 (44.4%) were females. Among the 168 parents with PD, 83 (49.4%) were females (mothers), which was not significantly different compared to the offspring generation (2-tailed Fisher exact p = 0.32).

Next, we assessed the association of mitochondrial haplogroups with the risk of maternal transmission of PD. The most common European mitochondrial haplogroups were defined as shown in Table 2. There were no significant differences in frequencies of any individual haplogroup among families in which the father had PD compared to families in which the mother had PD (Table 3).

**Table 3:** Association results for risk and gender of affected parent

	Fisher's Exact Test			McNemar's Test		Fisher's Exact Test		
**Allele**	**Cases**	**Controls**	**p**	**Risk**	**p**	**Affected Mother**	**Affected Father**	**p**

1719A	93.4%	95.9%	0.09	-	0.11	90.0%	91.0%	0.83

4580A	97.2%	97.4%	0.84	-	1.00	95.0%	98.5%	0.24

7028C	54.1%	55.3%	0.69	-	0.74	61.3%	53.7%	0.36

8251A	94.9%	95.1%	0.88	-	1.00	87.5%	95.5%	0.09

9055A	92.3%	90.2%	0.25	+	0.29	96.2%	89.6%	0.11

10398G	19.4%	20.9%	0.57	-	0.62	20.0%	20.9%	0.89

10400T	0.6%	0.4%	0.65	+	1.00	0.0%	1.5%	0.27

12308G	21.4%	23.3%	0.48	-	0.52	21.2%	23.9%	0.70

13368A	89.5%	91.2%	0.38	-	0.44	91.2%	92.5%	0.78

13708A	88.0%	86.8%	0.55	+	0.61	86.2%	91.0%	0.37

16391A	98.1%	97.4%	0.51	+	0.66	95.0%	98.5%	0.24

H	43.6%	42.9%	0.84	+	0.90	36.2%	46.3%	0.22

I	1.7%	2.4%	0.49	-	0.65	5.0%	0.0%	0.06

J	9.2%	10.9%	0.38	-	0.43	12.5%	7.5%	0.31

K	7.5%	9.4%	0.29	-	0.34	3.8%	10.4%	0.11

M	0.6%	0.4%	0.65	+	1.00	0.0%	1.5%	0.27

T	9.4%	8.3%	0.57	+	0.65	8.8%	6.0%	0.52

U	12.6%	13.0%	0.84	-	0.92	17.5%	10.4%	0.22

V	2.4%	2.4%	1.00	-	1.00	3.8%	1.5%	0.40

W	1.7%	1.7%	1.00	-	1.00	3.8%	1.5%	0.40

X	2.8%	0.6%	*0.01*	+	*0.02*	1.2%	7.5%	0.06

Other	8.5%	7.9%	0.72	+	0.81	7.5%	7.5%	0.99

IJK vs. HMTUVWX	18.4%	22.6%	0.11	-	0.12	21.2%	17.9%	0.61

JTUK vs. HIMVWX	31.2%	33.1%	0.53	-	0.57	35.0%	26.9%	0.29

JTIWX vs. HKMUV	24.8%	23.9%	0.76	+	0.82	31.2%	22.4%	0.23

When allele frequencies were compared between cases and controls (Table 3), haplogroup X showed a trend toward greater disease risk (p = 0.01); however, this association was not significant after controlling for multiple testing (even when only considering testing each of the ten haplogroups separately (rather than in combinations), which requires a Bonferroni correction of alpha = 0.005). The frequencies of haplogroup clusters previously reported in some studies to be associated with an increased (JT[31]; JTIWX[26]) or reduced (UKJT[27,40]) risk of PD, were not significantly different between PD and control subjects in the population of subjects reported here. There were no significant associations of the 10398G SNP or haplogroups (individually or in clusters) with the age of onset of PD (Table 4). When analyzing the mean age at onset within a nuclear family, carriers of haplogroup U tended to have an earlier PD onset (p = 0.03). A similar trend was seen for a SNP that tags haplotype U (12308G; p = 0.01). These associations were not significant after correcting for multiple testing.

**Table 4:** Association results for age at onset

	Age At Onset (cases)			Age At Onset (sibship^1^)		
**Allele**	**Carriers**	**Non-carriers**	**p**	**Carriers**	**Non-carriers**	**p**

1719A	61.7	63.2	0.52	61.8	64.4	0.16

4580A	61.8	60.4	0.61	62	61.1	0.76

7028C	61.2	62.4	0.21	61.5	62.5	0.28

8251A	61.9	59.2	0.20	62.1	59.5	0.20

9055A	62.0	59.8	0.22	62.1	60.2	0.26

10398G	60.3	62.2	0.11	60.6	62.3	0.15

10400T	57.7	61.8	0.48	60.2	62.0	0.75

12308G	60.2	62.2	0.08	59.8	62.5	*0.01*

13368A	61.6	63.7	0.17	61.7	63.8	0.17

13708A	61.8	62.0	0.86	62.0	61.9	0.98

16391A	61.9	58.9	0.39	62.0	58.3	0.26

H	62.2	61.5	0.49	62.4	61.6	0.41

I	59.6	61.8	0.54	59.3	62.0	0.45

J	61.4	61.8	0.80	61.7	62.0	0.89

K	59.6	62.0	0.18	60.0	62.1	0.22

M	57.7	61.8	0.48	60.2	62.0	0.75

T	63.7	61.6	0.20	63.6	61.8	0.23

U	60.4	62.0	0.26	59.4	62.3	*0.03*

V	60.9	61.8	0.77	61.3	62.0	0.83

W	60.2	61.8	0.67	58.9	62.0	0.38

X	65.2	61.7	0.45	67.1	61.8	0.06

Other	62.6	61.7	0.63	63.3	61.8	0.35

IJK vs. HMTUVWX	60.5	62.1	0.19	60.8	62.2	0.23

JTUK vs. HIMVWX	61.1	62.1	0.35	60.8	62.5	0.09

JTIWX vs. HKMUV	62.5	61.6	0.41	62.7	61.7	0.34

^1 ^Since all siblings share the same mitochondrial SNP alleles and therefore haplogroups, testing for association with the mean age at onset of all affected siblings in a nuclear family is a valid and more powerful test. The number of affected siblings in a family ranged from 1 to 5.

## Discussion

This study of the PROGENI subjects represents the first to report data on mitochondrial genetic variants in conjunction with maternal inheritance in a large group of familial PD cases, and fails to reveal a maternal bias in the inheritance of familial PD. The question of maternal inheritance in PD has been controversial, with some studies reporting a bias towards maternal inheritance in PD [5,6], and others failing to find this pattern [7,8]. Differences in methodologies and patient populations may contribute to the disparate results. De Michele et al[7] reported that secondary cases of 100 PD patients were more common in the paternal versus maternal line (70 versus 39). A questionnaire administered to 252 PD patients by Zweig and colleagues[8] identified 11 fathers and 5 mothers reported to have PD. Swerdlow and colleagues[5] subsequently addressed this issue in a larger set of 600 PD probands in their clinical database. They identified an affected parent in 13% of cases. Though 60% of the probands were male, only 42% of the affected parents were male, indicating an increased representation of females among parents with PD compared to the probands with PD (p < 0.005). Wooten et al[6] used a different approach, prospectively identifying families with an affected parent and multiple affected siblings. 57% of all PD cases in their database were male. The mother was the affected parent in all 5 of the first 5 such families, which represents a greater representation of females than expected if there was no gender effect (p < 0.03). Maher et al[41] reported that 60% of the probands among 948 consecutively ascertained PD cases were male whereas a greater percentage of affected parents were mothers compared to fathers (68 mothers versus 48 fathers; p = 0.0001 two-tailed Fisher exact p comparing male:female ratios in probands versus affected parents).

The current study failed to detect a significant bias towards maternal inheritance in familial PD, adding additional uncertainty regarding the role of inherited mtDNA variants in PD. A potential concern for this study is that the stronger genetic influence in familial PD cases might have led to a closer to 50:50 ratio of males to females in contrast to the male predominance for sporadic PD. However, this observation is unlikely to be a factor in this study as siblings and parents came from the same families. To improve the ability to detect an influence of mtDNA genetic variants, all families with known causative mutations in the *PARK2 *or *LRRK2 *genes (either two *PARK2 *mutations or one *LRRK2 *mutation), [42,43] were excluded from these analyses. Most of the samples were not screened for mutations in other PD-associated genes such as *PINK1 *and *DJ1*, but such mutations are relatively rare [44].

Mitochondrial genes are passed on from mothers to offspring but not from fathers to offspring. Therefore, to the degree that heritable mitochondrial genetic factors influence the risk of PD, there should be a greater risk of PD among offspring of women with PD compared to offspring of men with PD. However, alternative mechanisms also could result in a maternal inheritance bias. One example is epigenetic changes (imprinting) of autosomal genes. An additional potential mechanism is *in utero *environmental factors[45], as suggested by data demonstrating delayed loss of dopaminergic neurons following in utero exposure to lipopolysaccharide in rodents[46,47]. Another consideration is that historically women tend to live longer than men and are more likely to develop age-related diseases. Another possibility is that women affected with PD could be more likely to have a stronger genetic influence on the disease compared to men with PD. However, despite these additional possible causes of a maternal inheritance pattern in PD, we did not detect this pattern in our study population.

Indirect data has implicated mtDNA in the mitochondrial complex I defect in PD. "Cybrid" cell lines expressing mtDNA from PD patients manifest impaired mitochondrial complex I activity [9,10], suggesting that the complex I defect in PD results from stable differences in mtDNA. One possible explanation for these results from studies of cybrids despite a lack of consistent evidence for maternal inheritance in PD is that somatic mtDNA mutations rather than inherited mutations might account for the complex I defect in PD [48]. Heteroplasmic somatic mutations may be individually rare, but could accumulate to reach functionally significant levels in aggregate, though they would not be reliably detected with the genotyping methods used in the current study. Alternatively, the role of inherited mtDNA mutations, which can be sporadically expressed, may be different in sporadic PD compared to familial PD, with a greater influence of nuclear genetic factors influencing familial PD.

Another way to test a role for mtDNA variants in PD, in addition to testing for a maternal inheritance pattern, is to compare the frequencies of mtDNA variants between cases and controls. Prior studies of mitochondrial variants in PD have failed to identify clearly pathogenic mutations in the vast majority of patients [19,20], but these prior studies did not adequately address the possibility of a milder influence on PD risk from more common mtDNA variants. Our results add to the uncertainty regarding the previously reported association of the 10398G mitochondrial complex I gene polymorphism with a lower risk of PD[25,32]. Van der Walt and colleagues reported a reduced frequency of this polymorphism in Caucasian PD patients, suggesting a protective effect[32], and this inverse association was confirmed in a subsequent study in Spanish patients[25]. However, a previous study found no association of the 10398G variant with the risk of PD in the subset of Caucasian subjects in that study[19], and a later study in Italian subjects similarly detected no association of this variant with PD[24]. The current study, which included only Caucasian subjects, also failed to identify a significant association of the 10398G variant with the risk of PD, and furthermore failed to reveal an association of this variant with the age of onset of PD. Variability in the reported association of the 10398G variant with PD could relate to distinct subject populations across studies, and thus further studies are needed to determine if this polymorphism may influence the risk of PD in certain subsets of PD patients.

Reports of a possible association of haplogroups J and K with PD have been inconsistent [24,27,29-32]. Mitochondrial haplogroup clusters also variably have been reported to be associated with an increased (JT[31]; JTIWX[26]) or decreased (UKJT[27,40]) risk of PD, while others have failed to detect a significant association of UKJT[29]. In the current study of Caucasian familial PD subjects, we did not find evidence for an association of any mitochondrial haplogroup with the risk of PD or with age of onset of PD. Several factors, including distinct populations, different numbers of subjects, and distinct methods of analyses may contribute to the variability in results across studies. One major strength of the study is the one-to-one matching of cases and controls based on ethnicity (Figure 1). Population stratification is always a concern for genetic association studies, and if the frequency of specific haplogroups varies between Caucasians with different ancestries (e.g. Northern European versus Southern European versus Ashkenazi), then previous association results may be purely the result of admixture within the study population and therefore spurious.

## Conclusions

We did not detect a maternal bias in the inheritance of PD among PROGENI subjects, and failed to confirm previously reported associations of inherited mtDNA variants with PD risk. This suggests that other factors, possibly including somatic mtDNA mutations, may account for the complex I defect in PD.

## Competing interests

The authors declare that they have no competing interests.

## Authors' contributions

DKS conceived of the study, participated in its design, data analyses and interpretation, and drafted the manuscript; NP participated in the design of the study, performed the statistical analyses, and aided in interpreting the results and helped to draft the manuscript; DKK and MWP assisted in the design of the molecular studies and performed the laboratory experiments; CAH coordinated the recruitment, initial screening and follow-up of the subjects; AR participated in the design of the overall recruitment portion of the study and ensured regulatory oversight at the sites. RP participated as Chair of the Steering Committee; WCN assisted in planning the study and conducted the genotyping; TF conceived of the study, participated in its design and coordination and in analysis and interpretation of the results, and helped to draft the manuscript. All authors have read and approved the final manuscript.

## Pre-publication history

The pre-publication history for this paper can be accessed here:

http://www.biomedcentral.com/1471-2350/11/53/prepub
